# Simultaneous learning of individual microRNA-gene interactions and regulatory comodules

**DOI:** 10.1186/s12859-021-04151-2

**Published:** 2021-05-10

**Authors:** Michael Roth, Pranjal Jain, Jinkyu Koo, Somali Chaterji

**Affiliations:** 1 Google North America, San Francisco, USA; 2 Electrical Engineering, Indian Institute of Technology Bombay, Mumbai, India; 3 NVIDIA North America, Santa Clara, USA; 4 Agricultural and Biological Engineering, Purdue University, West Lafayette, IN USA

**Keywords:** Clustering, Bioinformatics, Regulatory comodules, miRNA-gene interaction

## Abstract

**Background:**

MicroRNAs (miRNAs) function in post-transcriptional regulation of gene expression by binding to target messenger RNAs (mRNAs). Because of the key part that miRNAs play, understanding the correct regulatory role of miRNAs in diverse patho-physiological conditions is of great interest. Although it is known that miRNAs act combinatorially to regulate genes, precise identification of miRNA-gene interactions and their specific functional roles in regulatory comodules remains a challenge. We developed Theia, an effective method for simultaneously predicting miRNA-gene interactions and regulatory comodules, which group functionally related miRNAs and genes via non-negative matrix factorization (NMF).

**Results:**

We apply Theia to RNA sequencing data from breast invasive carcinoma samples and demonstrate its effectiveness in discovering biologically significant regulatory comodules that are significantly enriched in spatial miRNA clusters, biological pathways, and various cancers.

**Conclusions:**

Theia is a theoretically rigorous optimization algorithm that simultaneously predicts the strength and direction (i.e., up-regulation or down-regulation) of the effect of modules of miRNAs on a gene. We posit that if Theia is capable of recovering known clusters of genes and miRNA, then the clusters found by our method not previously identified by literature are also likely to have biological significance. We believe that these novel regulatory comodules found by our method will be a springboard for further research into the specific functional roles of these new functional ensembles of miRNAs and genes,especially those related to diseases like breast cancer.

**Supplementary Information:**

The online version contains supplementary material available at 10.1186/s12859-021-04151-2.

## Introduction

Genes are distinct nucleotide sequences that contain the instructions for synthesizing proteins within cells. These instructions are turned into the actual protein that the gene *codes* for via RNA transcripts, and further translation into proteins, the latter in the case of protein-coding genes. One of the ways in which this activity is moderated is by interference from small non-coding RNA molecules called microRNA (miRNA). MiRNAs, genes, and their products all interact, forming complex *regulatory modules*. MiRNAs are short non-coding RNAs (of about 22 nucleotides) that regulate gene expression by both transcriptional and post-transcriptional mechanisms via binding to cognate messenger RNAs (mRNAs). Since the first miRNA lin-4 was discovered in 1993 [1], an increasing number of miRNAs have been found to affect a wide range of cellular and developmental processes through gene regulation [2]. Thus, accurately determining the miRNA targetome is crucial for understanding the role of miRNAs in various biological processes. However, most miRNAs’ specific functional roles and their combinatorial effects are still unclear.

*The miRNA interactome*: In order to identify potential miRNA-gene interactions, the first generation of computational methods relied mainly on complementarity between the miRNA seed region and the 3’-UTR section of mRNA, evolutionary conservation, and thermodynamic factors [3–6]. Sequence-based methods led to many false positives and some false negatives, and they are now primarily used as a tool to build putative interaction databases [7], such as in our own work [8–11].

*Mapping a dynamic interactome using context-specific interaction data*: While sequence information is static, expression profiles of miRNAs and genes are context-specific, providing useful clues on regulatory effects that may vary depending on conditions such as development or disease progression (temporal), and cell-type (spatial) context. Thus, context-dependent regulation can be studied by analyzing condition-specific or time-series expression data. Initial attempts to make use of expression data involved correlation analyses that measured the Pearson Correlation Coefficient (PCC) between miRNA and gene expression levels [12]. Although PCC can decide regulation strength and direction for the validated interacting pairs, the PCC value on its own can’t tell which pairs are interacting, since non-interacting pairs may show significant PCC values. In addition, the correlation coefficients for interacting pairs can be small, giving rise to high error rates [11]. These methods cannot adequately model the joint relationships between miRNAs and genes [13]. To model the combinatorial effects of multiple miRNAs on genes, multi-dimensional linear regression with regularizations (e.g., Lasso regression [14] and Elastic Net regression [15]) have been proposed. Unfortunately, they can only provide a sparse solution, i.e., a relatively small set of strong miRNA-gene interactions while disregarding the much more common subtle interactions [16], or they may have inaccurate estimates for weak interactions, further stymied by noisy or false interactions that can have a relatively high PCC, as seen in our recent work [11].

*Need for a modular and biologically interpretable framework*: Intuitively, miRNA-gene interactions can be better understood by considering regulatory comodules that group miRNAs and genes that collectively interact in the regulation process, as evidenced in emerging studies [17, 18]. This line of work typically integrates multiple genomic data sources, including sequence-based putative miRNA-gene interactions (putative interactions for short), protein-protein interactions (PPI), and miRNA and gene expression data. For example, SNMNMF [19] jointly analyzed miRNA and gene expression profiles in a non-negative matrix factorization (NMF) framework, in which matrix components were decomposed to provide information about regulatory comodules. To enhance the results, it integrated putative interactions and PPI simultaneously as regularization terms of the NMF problem. However, SNMNMF was designed to find the regulatory comodules only (i.e., grouping miRNAs and genes) and required additional steps to figure out the regulation strength of a particular miRNA-gene interaction. A regression-based model called PIMiM [20] handled this shortcoming by estimating the interaction strength by multiplying with module membership matrices. PIMiM reported better results than SNMNMF in discovering regulatory comodules [20], but its accuracy for estimating interaction strength is still lower than the state-of-the-art [11]. Meanwhile, both SNMNMF and PIMiM restrict their models to down-regulation only (anti-correlation between miRNA and gene expression), which is inconsistent with recent research results reporting that up-regulations also exist [21]. In fact, gene up-regulations are rampant in different cell phenotypes such as in cancer pathologies, an example being the up-regulation of flap endonuclease 1 (FEN1) in cancer progression [22] and up-regulation of the small GTP-binding protein, RhoA, in vascular hypertension [23]. The learned module membership matrices in our work are used to estimate individual miRNA-gene interactions. Similar to Tiresias [11], these interaction estimates are in turn used by a regression network along with expression data to find context-specific regulation strength *and* direction. Using the module membership matrices, which Tiresias does not deploy, Theia suppresses noise in the interaction edges better by disconnecting the miRNAs and genes that do not belong to at least one common regulatory comodule.

To summarize, our main contributions in this paper are as follows:

1. We develop a framework for simultaneous learning of regulatory comodules and miRNA-gene interactions, leveraging inter-dependency between diverse data sources. We synergistically associate the miRNA-gene ensembles with the individual interactions in a single framework such that optimizing one drives the other also to improve. Thus, we find that the accuracy in predicting the interaction profile of these miRNA is implicitly tied to the ability to model the miRNA and genes in these functionally meaningful mixed-membership modules.
2. Our method is able to accurately discover the regulatory comodules, achieving the ARI of up to 0.8 even at a biologically-plausible low regulation strength and outperforming SNMNMF and PIMiM significantly. With TCGA-BRCA data set, we show the comodules we found are significantly enriched in miRNA spatial clusters and gene ontology BP terms. From miRCancer, we also show that most of miRNAs (219 out of 319) and genes (88 out of 112) we found are cancer-related.
3. We assess the biological significance of miRNA modules through comparison with spatial miRNA clusters, cancer-implicated miRNA clusters, and miRNA modules with functional roles previously identified in literature. We assess the gene modules through Gene Ontology enrichment analysis, through the use of the Ingenuity Pathway Analysis software, and through a literature survey of the functional roles of gene modules.
  But since the ground truth does not exist for natural data, we further validate these systems through the use of synthetic data.
4. We consider both up-regulations and down-regulations in the miRNA-gene interactions within one unified framework. In doing so, we capture the more recent set of upregulated interactions that have been biologically validated.

## Background

*Non-negative matrix factorization (NMF)*: The non-negative matrix factorization (NMF) technique [24] was devised to factorize a non-negative matrix ${{\varvec{X}}}$ into two lower ranking matrices, a basis matrix ${{\varvec{W}}}$, and a coefficient matrix ${{\varvec{H}}}$, such that neither of these matrices contain negative elements. Such factorization can be achieved by minimizing the cost function as follows:

1 $$\begin{aligned} \min _{{{\varvec{W}}},{{\varvec{H}}}} \Vert {{\varvec{X}}}-{{\varvec{W}}}{{\varvec{H}}} \Vert , \text { subject to } {{\varvec{W}}}\ge 0, {{\varvec{H}}}\ge 0 \end{aligned}$$

The non-negativity of ${{\varvec{W}}}$ and ${{\varvec{H}}}$ guarantees that parts of the matrix can be combined additively to form a whole. Thus, NMF is a useful technique for obtaining a part-based representation of the data. NMF is inherently useful for the purpose of clustering because of the property that the the *j*th column of ${{\varvec{X}}}$ belongs to the *k*th cluster when ${\varvec{H}}_{kj}>0$.

*The NMF mechanics underlying* Theia: While the original use-case for NMF was dimensionality reduction, i.e., producing a low-dimensional feature representation of high-dimensional input data [24], recently, NMF has been found to be applicable to clustering by grouping elements that result in the same feature element [19, 25]. Inspired by this, we designed an NMF-based algorithm to produce a particular kind of grouping information-the comodule membership. Unlike recent manifestations of NMF-based clustering that factorize expression matrices, we apply this technique to putative miRNA-gene interactions and protein-protein interactions to assemble interacting miRNAs and genes into modules. Thus, the low-dimensional representation of the molecule interactions obtained via factorization becomes the comodule membership. NMF provides us with the ability to handle partially incorrect data. Furthermore, sparse representations and easily interpretable factors can be extracted using NMF. We leverage both these properties in our pipeline.

## Related work

*Comparison with SNMNMF*: Recognizing the regulatory comodules that model the groups of miRNAs and genes interacting collectively has greatly advanced our understanding of complex cellular systems. Representative work, SNMNMF [19] attempted to reconstruct the regulatory comodules based on the integration of multiple genomic data sources. However, fundamentally, it cannot provide the strength and the direction of the miRNA-gene interactions. We give the mathematical basis for this below.

Given expression profiles of miRNAs and genes, ${{\varvec{X}} \in {\mathbb {R}}^{N \times I}}$ and ${{\varvec{Y}} \in {\mathbb {R}}^{N \times J}}$, DNA-sequence-based putative interactions ${{\varvec{P}} \in \{0,1\}^{I \times J}}$, and protein-protein interactions ${{\varvec{Q}} \in \{0,1\}^{J \times J}}$, where *N* is the number of expression samples, *I* is the number of miRNAs and *J* is the number of genes, the core of SNMNMF is minimizing the following cost function:

2 $$\begin{aligned} \Vert {\varvec{X}}-{\varvec{A}}{\varvec{B}} \Vert +\Vert {\varvec{Y}}-{\varvec{A}}{\varvec{C}} \Vert -\lambda _1 \text {Tr}({\varvec{B}}{\varvec{P}}{\varvec{C}}^T)-\lambda _2\text {Tr}({\varvec{C}}{\varvec{Q}}{\varvec{C}}^T), \end{aligned}$$

where ${\varvec{A}} \in {\mathbb {R}}^{N \times M}$ is a new vector space with *M* denoting the number of modules, ${\varvec{B}} \in {\mathbb {R}}^{M \times I}$ and ${\varvec{C}} \in {\mathbb {R}}^{M \times J}$ are, respectively, new representations of ${\varvec{X}}$ and ${\varvec{Y}}$ on ${\varvec{A}}$, and $\text {Tr}(\cdot )$ denotes the trace of a matrix, the sum of the elements on the main diagonal. That is, SNMNMF integrates dynamic expression profiles of miRNAs and genes in a framework of multiple non-negative matrix factorization, and simultaneously integrates static supersets in a regularized manner. When trained, ${\varvec{B}}$ and ${\varvec{C}}$ matrices determine the comodule in such a way that miRNA and genes whose magnitudes on ${\varvec{B}}$ and ${\varvec{C}}$ are higher than thresholds in the same row belong to a common module.

However, by modeling the membership to the comodule *only by a threshold testing* on the non-negative elements of ${\varvec{B}}$ and ${\varvec{C}}$, i.e., grouping miRNAs and genes, this method does not provide the direction and strength of individual miRNA-gene interactions. Instead of factorizing ${\varvec{X}}$ and ${\varvec{Y}}$, Theia groups interacting miRNAs and genes by directly factorizing putative interaction data ${\varvec{P}}$ and ${\varvec{Q}}$, and refines the comodule memberships with additional modeling for the direction and strength of individual miRNA-gene interactions.

*Comparison with PIMiM*: PIMiM [20] alleviates the limitation of SNMNMF by estimating the interaction strength by multiplying the module membership matrices as follows:

3 $$\begin{aligned} \hat{{\varvec{y}}}_n = \varvec{\mu }-{\varvec{x}}_n {\varvec{U}}{\varvec{V}}^\intercal , \end{aligned}$$

where $\hat{{\varvec{y}}}_n\in {\mathbb {R}}^{1 \times J}$ is an estimate of ${\varvec{y}}_n$, the *n*th row of ${\varvec{Y}}$, ${\varvec{x}}\in {\mathbb {R}}^{1 \times I}$ is the *n*th row of ${\varvec{X}}$, and $\varvec{\mu }\in {\mathbb {R}}^{1 \times J}$ denotes the background mean of ${\varvec{y}}_n$ without regulation. The ${\varvec{U}} \in [0,\infty )^{I \times M}$ and ${\varvec{V}} \in [0,\infty )^{J \times M}$ are the module membership matrices whose elements larger than a threshold indicate the corresponding miRNA or gene (row indices) belong to a certain comodule (column indices). In PIMiM, the magnitude of an element in ${\varvec{U}}{\varvec{V}}^T$ is supposed to be proportional to the strength of a regulation relationship, if any. However, it still assumes the down-regulations only (i.e., an element in ${\varvec{U}}{\varvec{V}}^T$ is always non-negative), disregarding miRNA-modulated up-regulations. In addition, the magnitudes of elements in ${\varvec{U}}{\varvec{V}}^T$ are often non-zero even if they are smaller than a threshold and thus assumed to be non-interacting. These non-zero values act a noise when learning (3) by regression and thus reduce the accuracy of estimates for true interacting pairs of miRNAs and genes.

In contrast to PIMiM, Theia disconnects the non-interacting pairs from a regression relationship and thus suppresses the noise.

*Comparison with Tiresias*: On the other hand, Tiresias [11] models both up-regulations and down-regulations adopting what is called the regulation weight matrix as follows:

4 $$\begin{aligned} \hat{{\varvec{y}}}_n = \varvec{\mu }+{\varvec{x}}_n \left( {\varvec{S}}\bullet {\varvec{W}}\right) , \end{aligned}$$

where ${\varvec{W}}\in {\mathbb {R}}^{I \times J}$ denotes the regulation weight matrix whose elements model the regulation strength by their magnitude and regulation direction by their sign, ${\varvec{S}}\in [0,1]^{I \times J}$ is the true interaction indicator matrix whose element becomes 1 when an interaction is predicted and 0 otherwise, and the $\bullet$ operator denotes element-wise product. Tiresias jointly decides the elements of ${\varvec{S}}$ and ${\varvec{W}}$ so that $\hat{{\varvec{y}}}_n$ can be as close to ${\varvec{y}}_n$ as possible in a regression manner. This method was shown effective, achieving a higher F$^{1}$ score than the previous methods. However, Tiresias does not model the regulation comodules, studying the individual miRNA-gene interactions only.

Theia adopts the strategy that Tiresias used, i.e., Theia also models the interaction indicator matrix ${\varvec{S}}$ and the regulation weight matrix ${\varvec{W}}$, and by multiplying them together, Theia prevents pairs of miRNAs and genes that do not interact from affecting $\hat{{\varvec{y}}}_n$. This helps suppress the unwanted noise when learning $\hat{{\varvec{y}}}_n$ by a regression method. However, unlike Tiresias, Theia also models regulation comodules by additionally utilizing DNA-sequence-based putative interactions ${{\varvec{P}}}$ and protein-protein interactions ${{\varvec{Q}}}$, by which we subdivide miRNAs and genes into modules and allow them to interact only when they belong to at least one common module. By reducing this source of falsely predicted interactions, the accuracy of the interaction indicator matrix ${\varvec{S}}$ improves, and consequently, the accuracy of the regulation weight matrix ${\varvec{W}}$ as well.

## Materials and methods

### Data sources and preprocessing

We downloaded $N = 1161$ breast invasive carcinoma samples from the TCGA data portal, and filtered out miRNAs and genes with small expression values (less than 0.1). The mature data was extracted using Bioconductor packages [26]. As a result, we obtained a data set containing the expression profiles of miRNAs ${{\varvec{X}} \in {\mathbb {R}}^{N \times I}}$ and genes ${{\varvec{Y}} \in {\mathbb {R}}^{N \times J}}$ where $I = 979$ miRNAs and $J = 19258$ genes. The *n*th sample of miRNA expressions and the corresponding gene expressions, i.e., the *n*th rows of ${\varvec{X}}$ and ${\varvec{Y}}$, will be denoted ${\varvec{x}}_n=(x_{ni}) \in {\mathbb {R}}^{1 \times I}$ and ${\varvec{y}}_n=(y_{nj})\in {\mathbb {R}}^{1 \times J}$, respectively, where $x_{ni}$ is the expression of the *i*th miRNA and $y_{nj}$ is the expression of the *j*th gene, in the *n*th sample.

**Fig. 1:**
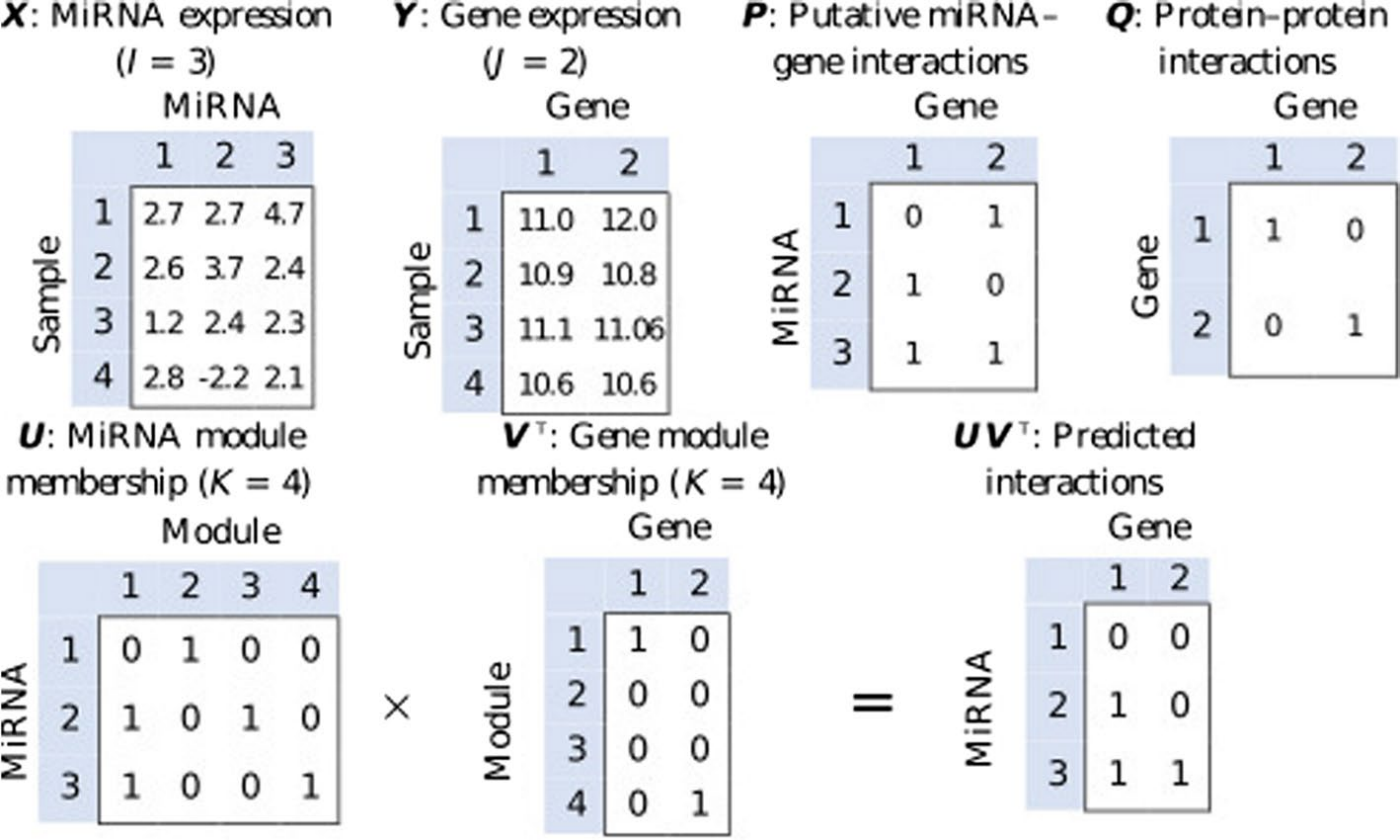
An example of expression profiles ${\varvec{X}}$ and ${\varvec{Y}}$ (when $I=3$ and $J=2$) are shown. Putative interaction matrices ${\varvec{P}}$ and ${\varvec{Q}}$ are shown alongside the expression matrices. Module membership matrices are represented by ${\varvec{U}}$ and ${\varvec{V}}$. By computing ${\varvec{U}}{\varvec{V}}^\intercal$, we can predict the interactions between miRNAs and genes

We constructed a putative interaction matrix, ${{\varvec{P}}=(p_{ij}) \in \{0,1\}^{I \times J}}$, from TargetScan [4] (release 7.1). The interactions represented by this matrix are *putative* because TargetScan is based primarily on target-site complementarity and suffers from a high false positive rate. Thus, $p_{ij} = 1$ suggests without any guarantee, that an interaction between the *i*th miRNA and the *j*th gene exists. It is possible that $p_{ij} = 0$ is a false negative; however, this is far less common [27, 28]. We constructed a matrix of proteinprotein interactions, ${{\varvec{Q}}=(q_{ij}) \in \{0,1\}^{J \times J}}$, from the Biological General Repository for Interaction Datasets (BioGRID) [29] (release 3.4.155). When the *j*th and $j^{\prime}$th genes interact, we set $q_{jj^{\prime}}=1$; otherwise, $q_{jj^{\prime}}=0$. Figure 1 shows a simple example to help understand our representation of data sources.

### Module membership and regulation weight matrices

The miRNA membership matrix ${\varvec{U}}=(u_{ik}) \in [0,\infty )^{I \times K}$ and gene membership matrix ${\varvec{V}}=(v_{jk}) \in [0,\infty )^{J \times K}$ model the regulatory comodules, where *K* is the number of modules. The matrix entries $u_{ik}$ and $v_{jk}$ denote the likelihood that the *i*th miRNA and *j*th gene belong to the *k*th module respectively (greater magnitude indicates a greater chance of belonging to the module). In Theia, a regulatory comodule is defined by miRNAs and genes that belong to a particular module in common. As seen in Fig. 1, when the *i*th miRNA and *j*th gene share membership in a particular module, the value of $({\varvec{U}}{\varvec{V}}^\intercal )_{ij}$, i.e., the (*i*, *j*) entry of ${\varvec{U}}{\varvec{V}}^\intercal$, is nonzero; thus, computing ${\varvec{U}}{\varvec{V}}^\intercal$ reveals the direct interactions between particular miRNAs and genes. Theia will utilize ${\varvec{U}}{\varvec{V}}^\intercal$ to decipher individual miRNA-gene interactions, represented by ${\varvec{W}}=(w_{ij}) \in {\mathbb {R}}^{I \times J}$ that we call the regulation weight matrix. The value of $w_{ij}$ estimates how strongly the *i*th miRNA regulates the *j*th gene (greater magnitude indicates stronger interaction). Further, the sign of $w_{ij}$ defines the direction of regulation, such that negative values indicate down-regulation and positive values indicate up-regulation.

### Overview

**Fig. 2:**
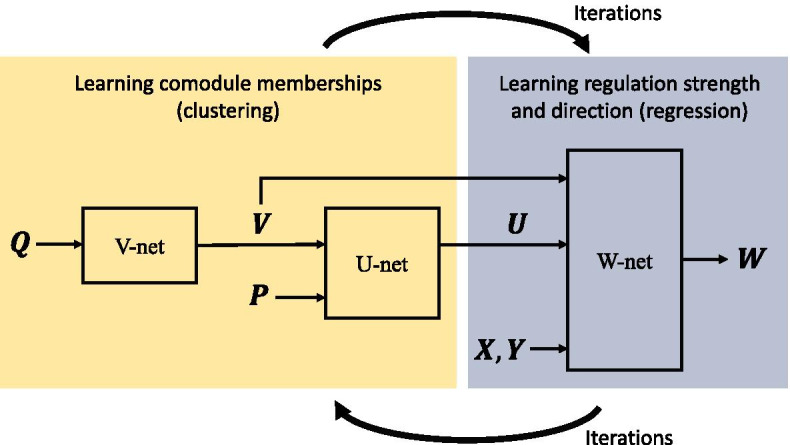
Overview of Theia. V-net learns the gene module membership, U-net learns the miRNA module membership, and W-net learns the regulation weights. This process repeats until the value of the cost function converges. All three sub-networks are trained together simultaneously and affect one another

Our goal is to simultaneously learn the module membership matrices ${\varvec{U}}$ and ${\varvec{V}}$, and also the regulation weight matrix ${\varvec{W}}$. Toward this end, we develop Theia, which is built from three networks: V-net, U-net, and W-net (see Fig. 2). The V-net first learns the gene module membership matrix ${\varvec{V}}$ from which the U-net then learns the miRNA module membership matrix ${\varvec{U}}$. By calculating ${\varvec{U}}{\varvec{V}}^\intercal$, the W-net predicts the true interaction matrix ${\varvec{S}}$ between miRNAs and genes, and from this, it learns the regulation weight matrix ${\varvec{W}}$. Subdividing miRNAs and genes into modules to allow them to interact only when they belong to at least one common module, the source of falsely predicted interactions is reduced. This improves the accuracy of the true interaction matrix ${\varvec{S}}$ and in turn, the accuracy of the regulation weight matrix ${\varvec{W}}$ as well.

All three sub-networks are trained together using ${\varvec{X}}$, ${\varvec{Y}}$, ${\varvec{P}}$, and ${\varvec{Q}}$ such that the inferencing of ${\varvec{U}}$, ${\varvec{V}}$, and ${\varvec{W}}$ affect one another. Such training is done by minimizing a single cost function (see (11)), adjusting ${\varvec{U}}$, ${\varvec{V}}$, and ${\varvec{W}}$ simultaneously. This will benefit the learning process because of the inherent dependencies between these biological data sources.

### V-net and U-net: non-negative matrix factorization

**Fig. 3:**
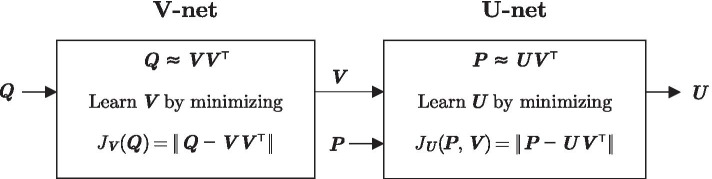
NMF to learn ${\varvec{V}}$ and ${\varvec{U}}$. V-net factorizes ${\varvec{Q}}$ into ${\varvec{V}}{\varvec{V}}^\intercal$. Given ${\varvec{V}}$ from V-net, U-net factorizes ${\varvec{P}}$ into ${\varvec{U}}{\varvec{V}}^\intercal$

As seen in Fig. 3, to incorporate the putative interaction data ${\varvec{P}}$ and proteinprotein interaction data ${\varvec{Q}}$ into our inference framework, we learn the module membership matrices by factorizing ${\varvec{P}}$ into ${\varvec{U}}{\varvec{V}}^\intercal$ (i.e., ${\varvec{P}}\approx {\varvec{U}}{\varvec{V}}^\intercal$) and ${\varvec{Q}}$ into ${\varvec{V}}{\varvec{V}}^\intercal$ (i.e., ${\varvec{Q}}\approx {\varvec{V}}{\varvec{V}}^\intercal$). A non-zero value of $({\varvec{U}}{\varvec{V}}^\intercal )_{ij}$ means that the *i*th miRNA and the *j*th gene interact belonging to a common module, and thus, ${\varvec{U}}{\varvec{V}}^\intercal$ should be similar to ${\varvec{P}}$. For a similar reason, ${\varvec{V}}{\varvec{V}}^\intercal$ should look like ${\varvec{Q}}$. Note that ${\varvec{P}}$ and ${\varvec{Q}}$ have a common factor ${\varvec{V}}$. We manage this constraint by first factorizing ${\varvec{Q}}$ in the V-net, and provide the result ${\varvec{V}}$ to the U-net, which in turn will learn ${\varvec{U}}$ by factorizing ${\varvec{P}}$. The factorizations performed by V-net and U-net minimize the respective cost functions,

5 $$\begin{aligned} J_{{\varvec{V}}}({\varvec{Q}}) = \left\| {\varvec{Q}} - {\varvec{V}}{\varvec{V}}^\intercal \right\| \end{aligned}$$

and

6 $$\begin{aligned} J_{{\varvec{U}}}({\varvec{P}}, {\varvec{V}}) = \left\| {\varvec{P}} - {\varvec{U}}{\varvec{V}}^\intercal \right\| . \end{aligned}$$

Because all the elements of ${\varvec{U}}$ and ${\varvec{V}}$ must be non-negative, the factorization should be accomplished using an NMF method [24]. Thus, we use the projected gradient descent algorithm [30] to minimize (5) and (6), which projects ${\varvec{U}}$ and ${\varvec{V}}$ to their nearest point in $[0,\infty )^{I \times K}$ and $[0,\infty )^{J \times K}$, respectively, whenever they contain negative quantities.

Note that unlike the common use case of NMF as a dimensionality reduction technique [19, 31], we adapt the algorithm for our specific use case. Conventionally, only one of the decomposed factors corresponding to representation of data matrix is useful and the other corresponding to the vector space (known as the basis matrix, typically with reduced dimensionality) does not play a role. Here, we decompose ${\varvec{P}}$ and ${\varvec{Q}}$, and the resulting factors ${\varvec{U}}$ and ${\varvec{V}}$ are all utilized as module membership matrices. It is also worth noting that unlike in PIMiM where zero elements of ${\varvec{Q}}$ are abandoned, we fully utilize all the information in ${\varvec{Q}}$.

Here, the module membership matrices ${\varvec{U}}$ and ${\varvec{V}}$ are mainly learned from the putative interaction information. However, later in W-net, these ${\varvec{U}}$ and ${\varvec{V}}$ will be refined in conjunction with expression profiles ${\varvec{X}}$ and ${\varvec{Y}}$.

### W-net: regression

Using ${\varvec{U}}{\varvec{V}}^\intercal$, W-net first computes ${\varvec{S}}=(s_{ij}) \in \{0, 1\}^{I \times J}$, which we call the true interaction matrix. The value of $s_{ij}$ is the probability that the *i*th miRNA truly regulates the *j*th gene, and it is defined as:

7 $$\begin{aligned} s_{ij} = \sigma \left( 2\beta ({\varvec{U}}{\varvec{V}}^\intercal )_{ij} - \beta \right) = \sigma \left( \beta (2({\varvec{U}}{\varvec{V}}^\intercal )_{ij} - 1\right) ), \end{aligned}$$

where $\sigma (z)=1/\left( 1+\exp (-z\right)$ denotes the sigmoid activation function, and $\beta$ is a scaling factor (which we set to $\beta =2$). In other words, $({\varvec{U}}{\varvec{V}}^\intercal )_{ij}$ is scaled by 2$\beta$ with a bias -$\beta$, in order to predict the probability of the true interaction between the *i*th miRNA and the *j*th gene. The value of $s_{ij}$ becomes nearer to 1 as the probability of the *i*th miRNA and the *j*th gene belonging to a common module increases, or equivalently, as the *i*th miRNA and the *j*th gene share an increasing number of modules in common. The *tanh* function is a scaled version of the *sigmoid* activation function, shifted vertically to adjust the range. The tanh activation can be rewritten as $tanh(z) = 2\sigma (2z)-1$. By introducing a bias of of -$\beta$, we shift the scaled activation horizontally. An advantage of the scaling factor, is that the gradient is larger than a regular sigmoid. Not only is this because of the multiplication by a 2$\beta$ factor, but also because the function becomes more sensitive to a change in the input. In our case, these two effects combined lead to faster convergence, assuming that the learning rate does not cause exploding gradients.

The regulation model $R({\varvec{S}},{\varvec{x}}_n,\varvec{\mu })$ produces an estimate of ${\varvec{y}}_n$, which is defined as:

8 $$\begin{aligned} \hat{{\varvec{y}}}_n = R({\varvec{S}}, {\varvec{x}}_n, \varvec{\mu }) = {\varvec{x}}_n\left( {\varvec{W}} \bullet {\varvec{S}} \right) + \varvec{\mu }, \end{aligned}$$

where $\hat{{\varvec{y}}}_n=({\hat{y}}_{nj}) \in {\mathbb {R}}^{1 \times J}$, $\varvec{\mu }=(\mu _{j}) \in {\mathbb {R}}^{1 \times J}$ with $\mu _{j}$ denoting the sample mean of the *j*th gene, and ${\varvec{W}} \bullet {\varvec{S}}$ denotes the element-wise product (also known as the Schur product) between two matrices ${\varvec{W}}$ and ${\varvec{S}}$. Thus, ${\hat{y}}_{nj}$ is expressed as:

9 $$\begin{aligned} {\hat{y}}_{nj} = \sum _{\forall i}w_{ij}s_{ij} x_{ni} + \mu _{j}, \end{aligned}$$

Namely, the $R({\varvec{S}},{\varvec{x}}_n, \varvec{\mu })$ is a regression network whose main purpose is to learn the regulation weight matrix ${\varvec{W}}$. The unknowns of the W-net (i.e., $w_{ij}$’s) are learned by mainly minimizing a cost function:

10 $$\begin{aligned} J_{{\varvec{W}}}({\varvec{X}}, {\varvec{Y}}, {\varvec{U}},{\varvec{V}}) =\frac{1}{N}\sum _{\forall n} \left\| \left( \hat{{\varvec{y}}}_n - {\varvec{y}}_n \right) \bullet \varvec{\sigma }^{-2} \right\| , \end{aligned}$$

where $\varvec{\sigma ^{-2}}=(\sigma _j^{-2}) \in {\mathbb {R}}^{1 \times J}$ with $\sigma _j^2$ denoting the sample variance of the *j*th gene. Scaling $({\hat{y}}_{nj} - y_{nj})$ by $1 / \sigma _j^2$ is intended to prevent a certain gene from dominating other genes in the regression due to its large expression magnitude.

Note that unlike in PIMiM [20], we utilize ${\varvec{U}}$ and ${\varvec{V}}$ to predict whether there are interactions (on or off), and separately adopt ${\varvec{W}}$ by which we can model the direction of regulations (up or down) as well as the strength. By taking the Schur product between ${\varvec{S}}$ and ${\varvec{W}}$ in (8), the *i*th miRNA and the *j*th gene whose $s_{ij}=0$ are disconnected in the regression network, and thus do not affect the minimization of the cost function in (10). This suppresses the unwanted interference when we learn ${\varvec{W}}$ by regression, and helps Theia decipher the small magnitudes of interactions, which are usually indistinguishable from noise in conventional regression methods. Note also that towards minimizing (10), the value of $s_{ij}$ is automatically learned.

### Combining U-net, V-net, and W-net

We have introduced three sub-networks of Theia, V-net, U-net, and W-net. These are dependent on one another and thus training one particular sub-network alone will not lead to the intended results. For example, the W-net requires ${\varvec{U}}$ and ${\varvec{V}}$ as its inputs, which are outputs of U-net and V-net. The U-net also needs ${\varvec{V}}$ that is the output of V-net. Training the V-net alone causes ${\varvec{V}}$ to be learned from ${\varvec{Q}}$ only, without considering ${\varvec{X}}$, ${\varvec{Y}}$, and ${\varvec{P}}$. Hence, a global optimization of all three networks is required, for which we find an appropriate objective function.

In order to train Theia such that all input data sets ${\varvec{X}}$, ${\varvec{Y}}$, ${\varvec{P}}$ and ${\varvec{Q}}$ are integrated, we minimize the following total cost function using the projected gradient descent:

11 $$\begin{aligned} J({\varvec{X}}, {\varvec{Y}}, {\varvec{P}}, {\varvec{Q}}) =&J_{{\varvec{W}}}({\varvec{X}},{\varvec{Y}},{\varvec{U}},{\varvec{V}}) + \lambda _1 J_{{\varvec{U}}}({\varvec{P}},{\varvec{V}}) + \lambda _2 J_{{\varvec{V}}}({\varvec{Q}}) + \frac{\lambda _3}{IJ} \sum _{\forall i, j}|w_{ij}|, \end{aligned}$$

where each $\lambda _n$ is a a weight that determines the relative importance of each term in the total cost function. The term $\sum _{\forall i, j}|w_{ij}| / (IJ)$ functions as a regularizer for the W-net in a similar manner as in the least absolute shrinkage selector operator (LASSO) regression. This helps Theia select significant interactions in ${\varvec{W}}$ while disregarding irrelevant ones.

**Fig. 4:**
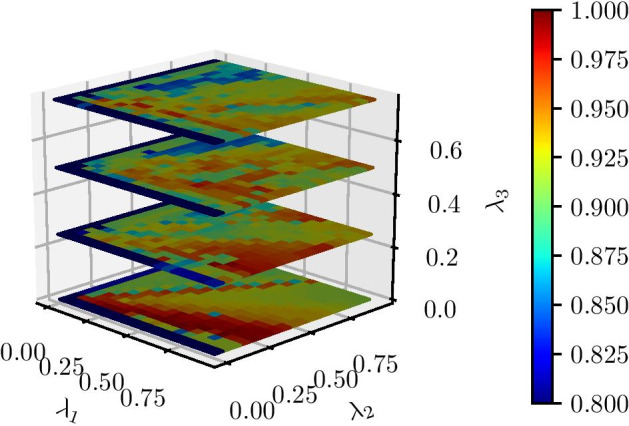
Adjusted Rand index for varying values of $\lambda _1$, $\lambda _2$, $\lambda _3$. $p_\text {signal}$ and $p_\text {fp}$ were held constant and set to 0.1 and 1 respectively (see the Synthetic data results section for the definitions of $p_\text {signal}$ and $p_\text {fp}$). Theia was run for each combination of $\lambda _1$, $\lambda _2$, and $\lambda _3$. The adjusted Rand index was calculated for each run, resulting in a three-dimensional matrix of results. This plot shows four horizontal slices of this matrix

We determined the values of $\lambda _1$, $\lambda _2$, and $\lambda _3$ to be 0.5, 0.5, and 0.25 by an exhaustive search as seen in Fig. 4, in which we search the range from 0 to 1 for each of the three aforementioned parameters. For each of the runs, we generated synthetic data (see the Synthetic data results section for a more in-depth discussion) and calculated the adjusted Rand Index. Note that Theia is not particularly sensitive to these parameters and performs relatively well (ARI near 1.0) even for sub-optimal configurations. We also tested the effect of varying $\lambda _n$ terms on the F$^{1}$ score. In this case, we saw that the variation was smaller than that of the ARI score and thus was not useful for determining the ideal set of parameters.

### Theia pipeline

In order to use Theia with the most recent biological data, the most recent putative interactions and proteinprotein interactions should be downloaded from databases such as TargetScan and BioGRID, respectively. MiRNA and gene expression data corresponding to the specific condition being studied must be downloaded as well. Then the training is initiated by executing the software program.

Once training is done, the *i*th miRNA and the *j*th gene are assigned to the *k*th regulatory comodules if $u_{ik} > T_{{\varvec{U}}}$ and $v_{jk} > T_{{\varvec{V}}}$, where $T_{{\varvec{U}}}$ and $T_{{\varvec{V}}}$ are cutoff thresholds. Note that each miRNA and gene may belong to multiple comodules, allowing for the identification of multiple functions. Theia will filter $w_{ij}$ by multiplying it with $s_{ij} p_{ij}$ because the value of $w_{ij}$ is hardly adjusted during training if $s_{ij} p_{ij}$ is near zero, and thus $w_{ij}$ is not particularly meaningful in this case. In order to detect individual miRNAgene interactions, Theia will compute the regulatory network edge matrix, ${{\varvec{E}} = (e_{ij}) \in {\mathbb {R}}^{I \times J}}$, defined as:

12 $$\begin{aligned} e_{ij} = s_{ij} p_{ij} w_{ij}. \end{aligned}$$

The edge matrix gives us an indication toward the existence of an interaction between *i*th miRNA and *j*th gene. To prevent spurious predictions, we apply a threshold $T_{{\varvec{W}}}$, where the interaction exists only if $e_{ij} > T_{{\varvec{W}}}$. The regulation strength and the direction (up or down) of the detected interactions are determined by the magnitude and sign of $w_{ij}$ respectively.

Our most novel algorithmic contribution involves learning the module membership matrices by decomposing ${\varvec{P}}$ and ${\varvec{Q}}$ using an NMF method, and utilizing them to predict true interactions. These interaction indicators are then used to disconnect non-interacting miRNAgene pairs in the regression network by which we can suppresses the unwanted interference when we learn the strength and direction of an regulation and improve the ability deciphering the small magnitudes of interactions.

## Results

In addition to recovering interactions between miRNAs and genes, our work aims to discover comodules composed of a cluster of miRNAs that work together to regulate genes that share a function. Ground truth of such modules do not directly exist. Thus, we are forced to rely on indirect evidence of the biological significance of our modules in the form of enrichment analysis and by searching for overlap between known gene clusters and our modules. The underlying intuition is that if Theia is capable of recovering known clusters, then the clusters found by our method *not* previously identified by literature are also likely to have biological significance.

We decided to set the dimension of the matrix factorization *K* to 195 because our data set contained a total of 979 miRNA and we expect approximately between 2 and 5 miRNAs per module ($979 / 5 \approx 195$) based on results from Zhang *et al*. [19] and the distribution of spatial miRNA cluster sizes. Additionally, the parameters $\lambda _1$, $\lambda _2$, $\lambda _3$, and $\beta$ were set to 0.5, 0.5, 0.25, and 2, respectively. We set $T_{{\varvec{U}}}=0.5$ and $T_{{\varvec{V}}}=0.25$ when deciding whether a particular miRNA or gene was part of a module. After applying Theia and discarding comodules containing less than two miRNAs or less than two genes, we have obtained 112 comodules with an average of 4.0 miRNAs and 102.2 genes per module.

Since we cannot be entirely sure of the biological significance of our comodules, we also validate our method by testing against synthetically generated data sets, in which we can control for some parameters, such as varying rates of false positives in the putative interactions and varying strength of regulation in the relationship between miRNA and gene expressions. This way, we can be more sure that our results on real data are meaningful despite being unable to verify this directly.

### The comodules are enriched in spatial microRNA clusters

Studies show that most miRNAs within 50 kilobase pairs (kb) tend to be co-expressed and regulate overlapping sets of target genes [32, 33]. This suggests that spatially clustered miRNA are likely to be functionally related or participate in cooperative regulation. Thus, one of the ways in which we evaluate the biological significance of the miRNAs within the comodules produced by Theia is by testing for spatial miRNA cluster enrichment.

Accordingly, we obtained miRNA sequences from the miRBase database [34–38] (release version 22) and grouped sequences within an inter-miRNA distance of 50 kb. This criterion resulted in a sample of 1479 clusters containing from 2 to 125 miRNAs. The average number of members per cluster is 3.2. Approximately half (748 out of 1479) of the miRNA clusters contained only two miRNAs. As mentioned earlier, this distribution of cluster sizes influenced our decision to set *K* to 195. The statistical significance (*p*-value) of the miRNA module’s enrichment in the spatial cluster was calculated using Fisher’s exact test. This statistic was transformed into a *q*-value by correcting for the false discovery rate [39]. Of the 112 comodules identified in this study, 37 are significantly enriched in at least one miRNA cluster (*q*-value < 0.05; see Table 1). All 112 comodules can be found in the additional files (see Additional file 1). For example, comodule 5 contains five miRNAs (*miR-449a*, *miR-449b-3p*, *miR-449b-5p*, *miR-449c-5p*, *miR-483-3p*), of which all but *miR-483-3p* belong to the miRNA cluster located on chromosome 5, band 11.2. Even though miRNAs within 50 kilobase pairs (kb) tend to be co-expressed and regulate overlapping sets of target gene, it is not necessary for comodules to only contain miRNAs within 50 kb; they may contain a mixture of miRNAs within 50 kb and further than 50kb. Our tool is an improvement over previous state-of-the-art methods, however, it is not perfect. Also, the miRBase database is growing as more discoveries are made. Perhaps our tool is predicting certain clusters not previously validated in the database, and some of these may be discovered in the future, while others may be false detections.

**Table 1:** Selected miRNA modules that are enriched in spatial miRNA clusters

Index	*q*-value	Overlap miRNAs	Loci
20	0.00	miR-512-3p, miR-515-3p, miR-516a-5p, miR-516b-5p, miR-517-5p, miR-517a-3p, miR-517b-3p, miR-518a-3p, miR-518a-5p, miR-518b, miR-518c-3p, miR-518c-5p, miR-518e-3p, miR-518f-3p, miR-518f-5p, miR-519a-3p, miR-519a-5p, miR-519c-3p, miR-520a-3p	19q13.42
5	2.13-6	miR-449a, miR-449b-3p, miR-449b-5p, miR-449c-5p	5q11.2
59	3.29-6	miR-489-3p, miR-653-3p, miR-653-5p	7q21.3
104	3.29-6	miR-221-3p, miR-222-3p, miR-222-5p	Xp11.3
49	7.83-6	miR-34b-3p, miR-34b-5p, miR-34c-5p	11q23.1
110	7.83-6	miR-301a-3p, miR-301a-5p, miR-454-3p	17q22
31	1.55-5	miR-1247-3p, miR-1247-5p	14q32.31
33	1.55-5	miR-10a-3p, miR-10a-5p	17q21.32
58	1.55-5	miR-552-3p, miR-552-5p	1q34.3
56	5.46-5	miR-132-3p, miR-212-3p	17q13.3
2	5.46-5	miR-105-5p, miR-767-3p, miR-767-5p	Xq28
68	5.46-5	miR-153-3p, miR-153-5p	7q36.3
78	1.59-4	miR-1-3p, miR-133a-3p	20q13.33
11	2.14-4	miR-15b-3p, miR-16-5p	3q25.33
76	2.72-4	miR-154-3p, miR-369-3p, miR-376a-3p, miR-409-5p, miR-411-5p, miR-487a-3p, miR-494-3p, miR-758-5p	14q32.31
62	2.96-4	let-7f-5p, miR-98-5p	Xp11.22
44	2.96-4	miR-199a-5p, miR-214-3p, miR-214-5p	1q24.3
95	6.54-4	miR-506-3p, miR-508-3p, miR-509-3p, miR-514a-3p	Xq27.3
35	7.51-4	miR-106a-3p, miR-20b-5p, miR-363-3p	Xq26.2
82	1.37-3	miR-192-3p, miR-194-5p	11q13.1

Index, the index of the comodule; *q*-value, the corrected *p*-value of enrichment; Overlap miRNAs, miRNAs in the module overlapping with the spatial cluster; Loci, the chromosomal location of the cluster

Quite a few of the miRNA modules found in this study are supported by existing literature. Notably, the miRNAs in comodule 20 are part of the C19MC cluster. Originally discovered by Bentwich *et al*. of Rosetta Genomics [40], the C19MC cluster spans 100 kb and yields 59 mature miRNAs, making it the largest cluster of miRNAs in the human genome [41]. The functional roles of the miRNA clusters in our modules are described exhaustively in Table 2.

**Table 2:** Summary of miRNA cluster functional roles based on literature survey

Index	Description	References
2	Aberrant expression of GABRA3 and the miRNAs it harbors (*miR-105*, *miR-767*) is reported in several tumor types. Furthermore, these miRNAs have been identified as protective in anaplastic gliomas	[42, 43]
5	The *miR-449* cluster regulates the Rb-E2F pathway, which controls the initiation of DNA replication and functions as a singal for inducing apoptosis	[44, 45]
11	*miR-15b* and *miR-16* target BCL2, which inhibits chemotherapeutic drug-induced apoptosis	[46]
20	Originally discovered by Bentwich et al. of Rosetta Genomics, the C19MC cluster spans 100 kb and yields 59 mature miRNAs, making it largest cluster of miRNAs in the human genome	[40, 41]
31	The *miR-1247* cluster directly targets SOX9, a transcription factor essential for cartilage formation and function and thus may be an important regulator of cartilage function. Increased expression of these mirNAs has also been shown to inhibit proliferation, tumorigenicity, colony formation and triggered G0/G1 cell cycle arrest in pancreatic cancer cells	[47, 48]
33	*miR-10a* is located in the Hox clusters of developmental regulators and was identified as a regulator of ribosome biogenesis and thus also global protein production. *miR-10a* and other miRNAs in the *miR-10* family are de-regulated in several types of cancer	[49]
44	*miR-199* and *miR-214* cooperatively function to differentiate mammalian skeletal precursor cells into osteoblasts or chondrocytes as well as develop muscles and the heart. These miRNAs are responsible for the development and progression of various cancers	[50]
49	The p53/*miR-34* pathway regulates cell death via apoptosis, thus the *miR-34* family acts primarily as a tumor suppressor	[51]
56	*miR-212/132* are tandem miRNAs that are responsible for the proper development, maturation and function of neurons. They are also known to function in inflammatory and immune processes	[52]
58	*miR-552* suppresses both transcription and translation of cytochrome P450 2E1, known to be important in the metabolism in ethanol and other low molecular weight chemicals	[53]
62	KMT2A upregulates the expression of the *let-7* family, which in turn inhibits cyclin D2. Inhibition of cyclin D2 in combination with up-regulation of these miRNAs mediate the suppression of cardiac hypertrophy	[54]
68	*miR-153* is negative regulator of both insulin and dopamine secretion. It is also both a suppressor and enhancer in tumor growth	[55, 56]
78	*miR-1/133a* are transcribed together but have opposing effects on myoblast proliferation differentiation. The former inhibits proliferation and promotes differentiation while the latter has the opposite effect. These miRNA are also known to be downregulated in bladder cancer and thus these miRNAs function as tumor suppressors	[57, 58]
82	*miR-192/194/215* play a role in kidney development and differentiation. These miRNAs are downregulated in clear cell renal cell carcinoma and thus are responsible for tumor-suppressor pathways	[59, 60]
104	*miR-221/222* are known to be potent regulators of p27^Kip1^, a cell cycle inhibitor and tumor suppressor	[61, 62]
110	*miR-130a/301a/454* promote the proliferation of colon cancer cells through inhibition of Smad4	[63]

Index, comodule index; Description, function according to literature abstract

### The comodules are enriched in known functional sets

In addition to testing our comodules for overlap with spatial miRNA clusters, we also performed functional enrichment analysis for genes in the identified comodules. Specifically, we looked for enrichment in Gene Ontology [64, 65] (GO) biological process (BP) terms. We filtered out GO terms with more than 300 associated genes or fewer than 5 genes. The thresholds used here for filtering GO terms, are the same ones used in SNMNMF [19]. The GO enrichment analysis was performed on each cluster using GOATOOLS [66], which computes the statistical significance of a module’s enrichment with Fisher’s exact test, with a *q*-value threshold of 0.05 (false discovery rate adjusted via the Benjamini-Hochberg procedure [67]). Also, note that the software was set such that term counts were not propagated to parents (propagate_counts=False).

Of the 112 gene modules identified by Theia, 48 (43%) have at least one overrepresented GO biological process (BP) term with an FDR-corrected *q*-value < 0.05. Alltogether, the modules are enriched in 302 unique GO biological processes. The most frequently enriched BP terms were cornification (14), cellular protein metabolic process (11), keratinization (8), fibrinolysis (5), muscle filament sliding (5), epidermis development (5), and platelet degranulation (5).

For comparison, when we performed the same test (three times) on the same number of randomly generated modules of the same size (112 total of size 102 genes), at most one module (1%) was enriched in at least one BP Term. This result is confirmed by the findings of Zhang *et al*. [19], who also performed GO enrichment on randomly generated modules and reported that only 2.4% of the modules were enriched in BP terms.

### The comodules are strongly implicated in cancer

Since our input data included the miRNA and gene expression profiles of breast cancer samples, we expected the identified comodules to be related to cancer. We validated this hypothesis by comparing the miRNAs in our modules to those in miRCancer, a miRNAcancer association database (release version October, 2017) [68]. This database contains a total of 767 oncomirs. Our 112 modules consisted of 319 unique miRNA of which 219 were found in the miRCancer database. Given that our input data consisted of 979 miRNA of which 289 are related to cancer, this ratio (219 / 289) is highly significant (*p*-value = 1.21-76). In addition, 88 of the 112 (79%) modules have at least two oncogenic miRNAs. Comodules were also analyzed via Ingenuity Pathway Analysis (IPA) software (QIAGEN Inc.). The software identified cancer as a top network in 77 of the 112 modules (69%). Results can be found in Table 3 with more detials in the additional files (see Additional file 2).

### The comodules form highly connected networks

The edges in IPA’s database of molecular interactions [69] connect genes on the basis of cause-effect relationships. Given that Theia groups genes that are related, we expect that dense graphs can be created from our generated gene modules. We used the default settings and found that in 88 of the 112 gene modules (79%) identified by our method, highly connected networks of genetic interactions could be constructed (score ≥ 35). Table 3 shows the top networks for some of the comodules. Among the top networks identified by the software were cancer (10), hereditary disorder (10), lipid metabolism (10), organismal development (10), cell morphology (12), cell-to-cell signaling and interaction (12), molecular transport (13), small molecule biochemistry (18), organismal injury and abnormalities (19).

**Table 3:** Functional analysis of selected gene module

Index	Top Networks	Cancer	*q*-value
1	Behavior, Endocrine System Development and Function, Cancer (35)	77/88	1.77-2
7	Connective Tissue Disorders, Developmental Disorder, Gastrointestinal Disease (46); Organismal Injury and Abnormalities, Renal Damage, Renal and Urological Disease (40)	107/122	2.85-2
9	Cancer, Organismal Injury and Abnormalities, Reproductive System Disease (57)	66/81	4.5-2
36	Cellular Movement, Immune Cell Trafficking, Connective Tissue Development and Function (43); Cellular Development, Cellular Growth and Proliferation, Connective Tissue Development and Function (43)	110/138	1.53-2
61	Cell Death and Survival, Cellular Compromise, Cell-To-Cell Signaling and Interaction (50); Endocrine System Disorders, Gastrointestinal Disease, Immunological Disease (37); Cell-To-Cell Signaling and Interaction, Hematological System Development and Function, Immune Cell Trafficking (35); Inflammatory Response, Cell Death and Survival, Cellular Compromise (35)	183/195	1.36-7
63	Lipid Metabolism, Small Molecule Biochemistry, Connective Tissue Development and Function (38); Cardiovascular Disease, Cell Death and Survival, Connective Tissue Disorders (38)	137/157	6.42-3
67	Cell Morphology, Cellular Function and Maintenance, Molecular Transport (46); Cancer, Connective Tissue Disorders, Organismal Injury and Abnormalities (41)	98/123	1.29-3
90	Connective Tissue Development and Function, Skeletal and Muscular System Development and Function, Tissue Development (36)	68/76	9.59-2
93	Hematological System Development and Function, Lymphoid Tissue Structure and Development, Tissue Morphology (40); Cancer, Dermatological Diseases and Conditions, Hematological Disease (38)	78/86	4.77-4
109	Cancer, Connective Tissue Disorders, Organismal Injury and Abnormalities (45)	84/102	1.52-3
110	Behavior, Cell-To-Cell Signaling and Interaction, Cellular Growth and Proliferation (40); Carbohydrate Metabolism, Cellular Function and Maintenance, Small Molecule Biochemistry (40)	85/101	1.47-2

Index, comodule index; Top Networks, top biological networks as identified by IPA software. Parenthesized value is the negative log of Fisher’s exact test *p*-value; Cancer, number of cancer-related genes within this comodule according to IPA software compared to total number of genes; *q*-value, multiple-test-corrected *p*-value of enrichment in cancer genes as reported by software

### Theia can recover validated miRNA-gene interactions

We also evaluate Theia’s ability to discover individual miRNA-gene interactions. For this experiment, we downloaded the list of experimentally-validated interactions from miRTarBase (release 6.0) [70]. To increase the confidence of the ground-truth interactions, we filtered the list of interactions with $|PCC|\ge 0.001$, $|PCC|\ge 0.01$, and $|PCC|\ge 0.1$. Note that we use absolute value of *PCC* here because positive values indicate up-regulation and negative values indicate down-regulation. This process resulted in 114, 108, and 45 validated interactions respectively. Note that the absence of an interaction in miRTarBase for the other pairs of miRNAs and genes does not necessarily mean that these pairs do not actually interact. Some of these pairs may indeed interact but are not yet validated by experiments. Thus, we cannot evaluate the precision and recall of Theia because non-validated interactions would be incorrectly counted as false positives. Instead, we focus on evaluating how well Theia can recover the validated interactions by computing the *detection rate*, which we define as the ratio of detected interactions to the total number of validated interactions (after filtering).

**Fig. 5:**
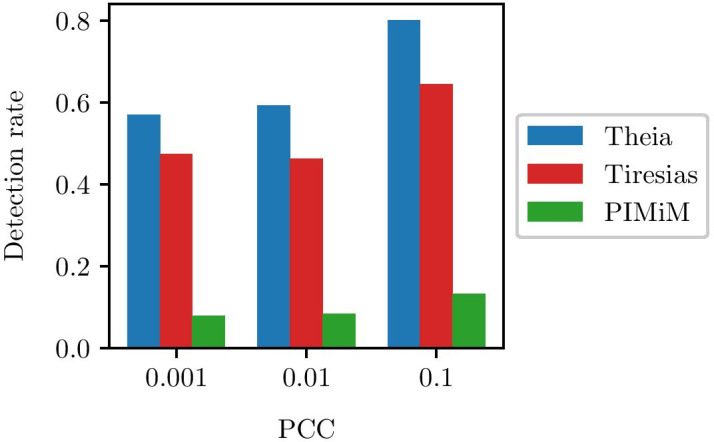
Detection rate when validated interactions are filtered by *PCC* thresholds. Note that during filtering we use |*PCC*| because positive values indicate up-regulation and negative values indicate down-regulation. Theia achieves the detection rate up to 0.8 depending on the strength of regulations, which is vastly superior to Tiresias and PIMiM. In all cases, we set $T_{{\varvec{W}}}=0.001$

In Fig. 5 we can see that Theia’s detection performance is naturally dependent on the regulation strength. Interactions are easier to detect when they are strong ($|PCC|\ge 0.1$); thus, both Tiresias and Theia suffer when weaker interactions are considered as well ($|PCC|\ge 0.001$). The detection rate that Theia achieves is 0.8 when $|PCC|\ge 0.1$ and 0.57 when $|PCC|\ge 0.001$. This is significantly higher than the detection rate of our competitors. In the same conditions, Tiresias [11] achieves at most 0.64 and PIMiM [20] obtains at most 0.13. PIMiM was not originally designed to consider regulation direction and always assumes down-regulation. To level the playing field, we advantaged PIMiM by giving it perfect knowledge of the regulation direction. That is, when PIMiM predicts a true interaction, we count it as a true positive regardless of its direction. Despite providing a similar advantage to SNMNMF, its detection rate was near zero and we omit its results here.

*Evaluation using cross validation* We evaluate Theia using ten-fold cross validation similar to what has been done in previous studies [71, 72]. The experiments have been performed using experimentally-validated interactions from the miRTarBase (release 6.0) [70]. In the ten-fold cross validation, all experimentally-validated interactions are randomly divided into ten-folds. For each round, one set is held-out and used for testing while the rest are used for training. The corresponding predicted result of test samples is considered as true positive (TP) when the predicted relevance score is greater than the threshold. Otherwise, false negative (FN). Similarly, for the unknown miRNA-disease interactions, the corresponding predicted result is considered as false positive (FP) when the predicted relevance score is greater than the threshold. Otherwise, true negative (TN). The AUC for each fold is calculated and the mean of these values is taken to get the mean AUC over ten folds. The training procedure remains identical except for a change in one of the inputs: the putative matrix ${{\varvec{P}}=(p_{ij}) \in \{0,1\}^{I \times J}}$, constructed from TargetScan [4] (release 7.1) is replaced by a matrix ${\varvec{P^{\prime}}=(p^{\prime}_{ij}) \in \{0,1\}^{I \times J}}$ constructed from the miRTarBase (release 6.0) [70]. All three sub-networks are trained together using ${\varvec{X}}$, ${\varvec{Y}}$, ${\varvec{Q}}$, and $\varvec{P^{\prime}}$. The mean AUC across ten folds is calculated for Theia, Tiresias [11] and PIMiM [20] and found to be 0.9294, 0.8536 and 0.6137 respectively.

### Evaluating Theia with synthetic data

Existing literature has only discovered a small fraction of the interacting miRNA-gene pairs. Target genes for rice miRNAs have been mainly predicted by computational approaches, and only a small fraction of targets has been experimentally validated [73]. Several high-throughput crosslinking-immunoprecipitation (CLIP) approaches have been reported to produce a high number of false negatives [4]. Furthermore, the detailed combinatorial roles of most miRNAs and genes are still unclear. This fact makes real datasets inadequate for computing evaluation metrics, for which knowledge of the ground truth is required. Consequently, we synthetically generate miRNA-gene interactions, regulatory comodules, miRNA and gene expressions, putative interactions, and protein-protein interactions. With this data where we can control ground truth, we evaluate the efficacy of Theia with varying rates of false positives in the putative interactions and varying ratios of signal to noise in the miRNA/gene expression.

*The need for synthetic datasets* Previous works focus on evaluating their methods on biological datasets. Out of previous works SNMNMF, PIMiM and Tiresias, only previous state-of-the-art Tiresias [11] makes use of synthetic data. However, in contrast to Tiresias, we evaluate the methods in our work on a much larger set of synthetic datasets. Figure 1 gives an example of matrices ${\varvec{X}}$ and ${\varvec{Y}}$ from which the PCC values are calculated. The sequences derived from these matrices, for an MiRNA and a gene, are used in this calculation, with each term corresponding to a sample; the number of terms is equal to the number of samples ${\varvec{N}}$. In this work, while generating synthetic datasets, we vary parameters $p_\text {signal} \in \{0, 0.05, 0.1, \dots , 1\}$ and $p_\text {fp} \in \{0, 0.05, 0.1, \dots , 2\}$. This gives rise to 861 combinations, since there are 21 possibles values of $p_\text {signal}$ and 41 possible values of $p_\text {fp}$. Table 4 shows us the effect that varying $p_\text {signal}$ has on the PCC values, and hence on the dataset matrices ${\varvec{X}}$ and ${\varvec{Y}}$. Even if we assume that synthetic datasets match each other closely for some range of $p_\text {signal}$, this range is small compared to the range of values in which we vary $p_\text {signal}$; observe in Table 4 that the change in PCC values is appreciable as we vary $p_\text {signal}$. Another question which arises is how closely the TCGA BRCA dataset matches the synthetic datasets. Even if we assume that for a certain pair of $p_\text {signal}$ and $p_\text {fp}$ the TCGA BRCA dataset matches the corresponding synthetic dataset, it is impossible for the TCGA BRCA to be matched closely to all synthetic datasets used, as this would contradict the observations of Table 4. In fact, in the case the TCGA BRCA dataset matches a synthetic dataset, it will be related to only a very small percentage of all the synthetic datasets used in this study. In this way we ensure two things; the availability of a ground truth to compare the predictions made by Theia with, and the evaluation of Theia on a large number of datasets. Both these factors reinforce the applicability of Theia to a larger range biological contexts.

*Synthetic data generation* Several studies indicate that the sizes of clusters of functionally related genes are distributed according to a positively skewed distribution. That is, the vast number of genes sets are relatively small, while a few are much larger [74]. One can also arrive at this conclusion from the perspective of genetic hubs, small class of genes that affect many different biological pathways [75]. These hubs tend to have a high level of connectivity in biological networks, and this means that they tend to be a part of large-sized modules and also appear in a large number of modules. On the other hand, the more common non-hub genes form smaller modules and appear in a few modules. Likewise, we see a similar skewed distribution in spatial miRNA clusters. The distribution as well as the distribution of GO BP term module sizes can be found in more detail (see Additional file 4). For these reasons, we generated comodules such that the number of members per module followed a positive-skewed distribution. In order to reflect the high connectivity of the genetic hubs, we also model the number of modules per miRNA/gene as a positive-skewed distribution. More details can be found in the additional files (see Additional file 3)

In detail, we generate the module membership matrices ${\varvec{U}}$ and ${\varvec{V}}$ such that their elements are either 0 or 1. The number of nonzero elements in each column of ${\varvec{U}}$ and ${\varvec{V}}$ (which represents the number of miRNAs or genes in a module) is distributed according to the skew normal distribution, $\mathcal {SN}(\xi , \omega , \alpha )$, with location parameter $\xi = 1$, scale parameter $\omega = 1$, and shape parameter $\alpha = 5$. In addition, the number of nonzero elements in each row of ${\varvec{U}}$ and ${\varvec{V}}$ (which represents the number of modules in which a particular miRNA or gene has membership) is also distributed according to the skew normal distribution, $\mathcal {SN}(\xi , \omega , \alpha )$, with $\xi =D/K$, $\omega = 10$, $\alpha = 5$ ($D = I$ for ${\varvec{U}}$ and $D = J$ for ${\varvec{V}}$).

The ground truth interactions between miRNAs and genes, ${\varvec{G}}=(g_{ij}) \in \{-1,0,1\}^{I \times J}$, is the product of the miRNA and gene module membership matrices. Thus, $g_{ij}$ is non-zero if the *i*th miRNA and the *j*th gene share at least one module in common. A majority (controlled by $p_\text {down}$) of the non-zero elements are made negative (representing a down-regulation). Precisely, we define ${\varvec{G}}$ as follows:

13 $$\begin{aligned} g_{ij} = \min (({\varvec{U}}{\varvec{V}}^\intercal )_{ij}, 1) (2b_{ij} - 1), \end{aligned}$$

where $$\text {Pr}(b_{ij} = 0) = 1 - \text {Pr}(b_{ij} = 1) = p_\text {down}$$.

The miRNA expression, ${{\varvec{X}} \in {\mathbb {R}}^{N \times I}}$, and gene expression, ${{\varvec{Y}} \in {\mathbb {R}}^{N \times J}}$, are distributed normally. Each sample of expression data is generated as follows:

14 $$\begin{aligned} x_{ni}&\sim {\mathcal {N}}\left( \mu _x, \sigma _x^2\right) \end{aligned}$$

15 $$\begin{aligned} y_{nj}&\sim {\mathcal {N}}\left( \mu _y + p_\text {signal}\sum _{\forall i} g_{ij}x_{ni}, \sigma _y^2\right) , \end{aligned}$$

where $\mu _x$ and $\mu _y$ are the average miRNA and gene expression levels respectively when there is no regulation. The parameters $\sigma _x^2$ and $\sigma _y^2$ are the expression level variances. The value of $p_\text {signal}$ controls the strength of the effect of the miRNA expression level on the gene expression level. By increasing this signal, the modules embedded in the expression data become easier to extract, while decreasing the signal results in the modules becoming obscured by the variance. The correspondence between $p_\text {signal}$ and the PCC is shown in Table 4.

**Table 4:** PCC corresponding to $p_\text {signal}$

$p_\text {signal}$	0.001	0.005	0.01	0.05	0.1	0.5
PCC	0.087	0.089	0.091	0.113	0.159	0.374

The PCC value shown here is the average of top 10 PCC values in interacting pairs of miRNAs and genes, generated with $p_\text {down} = 0.8$, $\mu _x = 3$, $\mu _y = 10$, and $\sigma _x^2 = \sigma _y^2 = 1$

Putative miRNA-gene interactions ${\varvec{P}}$ are generated:

16 $$\begin{aligned} p_{ij} = \max \left( g_{ij}, b^{\prime}_{ij}\right) , \end{aligned}$$

where $\text {Pr}(b^{\prime}_{ij} = 1) = 1 - \text {Pr}(b^{\prime}_{ij} = 0) = p_\text {fp}\sum _{\forall i,j}g_{ij}/(IJ)$. The parameter $p_\text {fp}$ controls the number of false positive interactions relative to the density of ${\varvec{G}}$. For example, when $p_\text {fp} = 1$, the density of ${\varvec{P}}$ is (roughly) doubled and approximately half of the interactions will be false positives. When $p_\text {fp} = 2$, the density is (roughly) quadrupled and approximately three-quarters of the interactions will be false positives, and so on. The purpose of this relative false positive rate is to make the effect of $p_\text {fp}$ independent of the dimensions of *G*.

Lastly, the protein-protein interactions ${\varvec{Q}}$ are generated:

17 $$\begin{aligned} q_{ij} = \min \left( ({\varvec{V}}{\varvec{V}}^\intercal )_{ij}, 1\right) . \end{aligned}$$

That is, $q_{ij} = 1$ if $({\varvec{V}}{\varvec{V}}^\intercal )_{ij} \ge 1$, and $q_{ij} = 0$ otherwise.

*Synthetic data results* All parameters for Theia were kept the same as in the biological dataset evaluation, except both the cutoff for ${\varvec{U}}$ and ${\varvec{V}}$ were set to 0.5. Comodules were generated according to the previously described procedure with the following parameters: $N = 1000$, $K = 10$, $I = 50$, $J = 500$, $\mu _x = 3$, $\mu _y = 10$, and $\sigma _x^2 = \sigma _y^2 = 1$.

In order to measure the similarity between modules discovered by Theia and the true modules, which we know in the case of the synthetic data, we use the adjusted Rand index (ARI). The basic Rand index (RI) computes a similarity measure between two clusterings by considering all pairs of elements and counting pairs that are assigned to the same cluster and dividing this value by the total number of pairs. However, the RI does not account for chance, i.e., two clusterings that are similar purely by chance. The likelihood of such chance placements is higher when there is a small number of clusters or a small number of elements or both. Thus, instead of the RI, we decide to use the ARI, which adjusts the index value to account for the expected similarity between the clusterings. The ARI lies between -1 and 1. Random clusterings have an ARI close to 0 while 1 stands for perfect match, and an ARI less than zero represents a worse-than-random clustering.

To evaluate Theia’s ability to recover the true miRNA-gene interactions, we use the F$^{1}$ score. If both the interaction and the direction of the interaction were correctly predicted, then this was considered a true positive. On the other hand, if an interaction was predicted when no interaction existed, this was considered a false positive. But if the lack of an interaction was correctly predicted, this was considered a true negative. The remaining cases were considered false negatives.

**Fig. 6:**
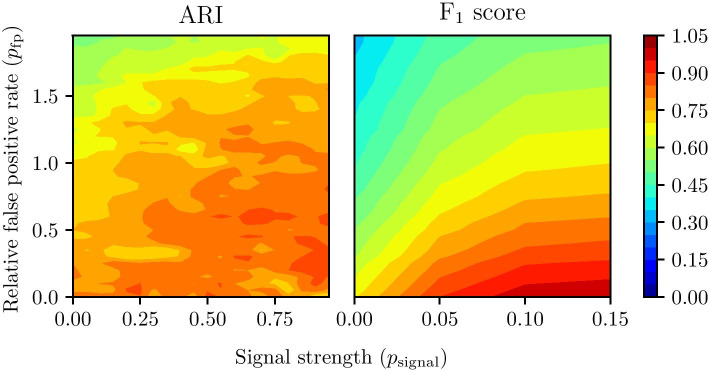
Performance of Theia on synthetic data. ARI of similarity between true modules and modules discovered by Theia, and the F$^{1}$ score of Theia recovering the true interactions. The *x*-axis indicates the regulation strength and the *y*-axis indicates the relative false positive rate in the putative miRNA-gene interactions. The *x*-axis of the F$^{1}$ score ends at 0.15 because the flat trend seen in this plot continues

The ARI and the F$^{1}$ score were computed for every combination of $p_\text {signal} \in \{0, 0.05, 0.1, \dots , 1\}$ and $p_\text {fp} \in \{0, 0.05, 0.1, \dots , 2\}$. A Gaussian filter with $\sigma = 0.5$ was applied to the 40 by 20 matrix of results to reduce the noise. Figure 6 shows the resulting contour plot. We can see that Theia can achieve an ARI near 0.9 when $p_\text {fp}=0$ and $p_\text {signal}=0.5$. This tells us that with no false positives in ${\varvec{P}}$ and a high signal strength, Theia can recover the ground-truth comodules almost perfectly. In the most biologically-plausible region (i.e., small PCC values, typically less than 0.1, which correspond to the region around $p_\text {signal}=0$), Theia can still achieve ARI ranging from 0.5 to 0.8, depending on $p_\text {fp}$. This is significantly better than existing solutions as we will see later. We can also see that Theia can achieve an F$^{1}$ score of up to 0.7 even when $p_\text {signal}=0$. This is the benefit of Theia’s use of ${\varvec{P}}$ and ${\varvec{Q}}$, from which we can learn much about interactions even without using expression data. With a high $p_\text {signal}$ and a low $p_\text {fp}$, Theia can perfectly recover true interactions (F$^{1}$ = 1).

The fact that Theia performs so well on synthetic data without the need for additional tuning of the hyper-parameters is suggestive of two facts. First, this indicates that Theia can easily adapt to different data sets, making this algorithm useful for a wide array of patho-physiological conditions. Second, this result validates the biological significance of our synthetic data generation procedure. Thus, our data generation algorithm can confidently be used to evaluate the effectiveness of future methods within this class of algorithms. This contribution is especially significant in the domain of computational genomics because the availability of high-quality ground truth data sets is often limited.

### Comparison with other methods

Unlike other recent methods for miRNA-gene regulatory comodule identification, Theia learns the comodules along with the regulation strength represented by ${\varvec{W}}$ in the regression network. This feature helps improve the accuracy of the comodules, since the regression network is mutually related with the comodules, and thus minimizing the cost of the regression network, which is contributed proportionally to ${\varvec{W}}$ by each interacting miRNA-gene pair, forces the module clustering as well to improve.

**Fig. 7:**
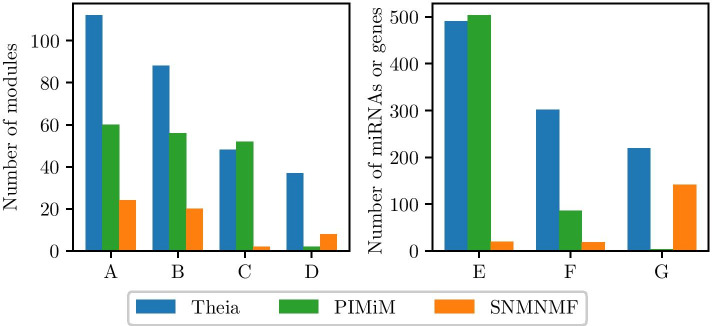
Metrics calculated using Theia, PIMiM, and SNMNMF on TCGA-BRCA data set. Metrics (B–G) are influenced by the total number of comodules discovered (A). The bars depict (A) the total number of comodules discovered by each method, (B) the number of modules with at least two oncomirs, (C) the number of modules enriched in at least one GO BP term, and (D) the number of modules enriched in spatial miRNA clusters, (E) the total number of GO BP terms that were enriched in the modules discovered by the method, (F) the total number of unique GO BP terms, and (G) the total number of oncomirs in the miRNA modules discovered by each method

To demonstrate that Theia’s approach can indeed improve the accuracy of comodules, we compare Theia with SNMNMF [19] and PIMiM [20] using TCGA-BRCA data set. For this experiment, the number of comodules *K* was set to 195 (which is roughly equal to #|miRNAs|/5) for all three methods. We set the weight parameters for SNMNMF $\lambda _1$, $\lambda _2$, $\gamma _1$, and $\gamma _2$ to 0.0001, 0.01, 20, and 10, respectively because these were found to be optimal in [19]. By following the procedure as described in the Supplementary Material in [19], we determined the optimal SNMNMF threshold *T* to be 1 because this value yielded the highest ratio of modules enriched in GO BP terms to total modules as compared to other values of *T* in the range 1 to 10. The regularization and weight parameters of PIMiM, $\alpha$, $\beta$, $C_1$, and $C_2$ were optimized by performing an iterative line search to determine the values of these parameters using the F$^{1}$ score as the target function to optimize (as described in [20]). The optimal parameters were found to be 1, 0.5, 3, and 3 respectively. The results of a comparative analysis can be seen in Fig. 7. In this figure, the metrics are influenced by the number of comodules predicted by each method. Note that some of the comodules predicted by the methods may not be functionally valid, and these metrics are affected by these errors.

We can see that Theia outperforms SNMNMF significantly in all respects. Although a slightly greater proportion of PIMiM’s modules were enriched in at least one GO BP term (C), we can attribute this to a large degree of inter-module overlap, as evidenced by the low number of unique GO BP terms (F). That is, there is a large amount of duplication within PIMiM’s modules causing causing (C) and (E) to be inflated.

The fact the modules discovered by Theia were enriched in such a large and varied group of GO terms indicates that our solution was able to cluster many miRNAs and genes according to their function, rather than just the most obvious relationships. Theia also had great success in constructing miRNA modules that overlapped with spatial miRNA clusters compared to competing solutions; 37 of Theia’s modules were enriched in these spatial clusters while less than 10 of SNMNMF’s and PIMiM’s modules were enriched in this way. Finally, many of the miRNAs in our modules are known to be associated to cancer. This result seems to suggest that our method is truly context sensitive in that it can identify regulatory comodules that are related to the patho-physiological condition from which the RNA expression data originated.

**Fig. 8:**
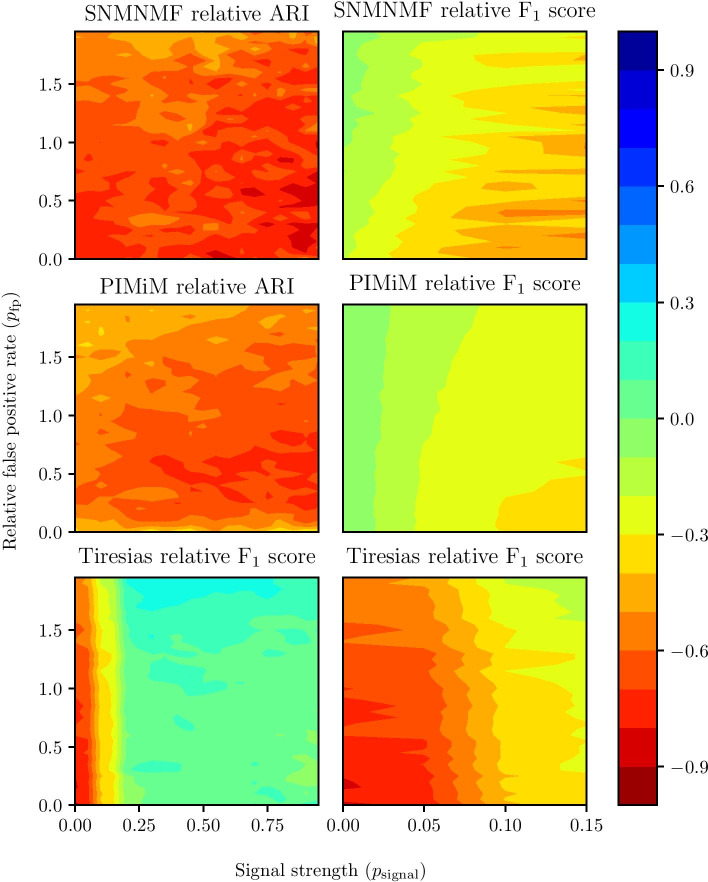
ARI and F$^{1}$ score comparison between Theia, SNMNMF, PIMiM, and Tiresias. The ARI and F$^{1}$ score of SNMNMF and PIMiM are compared relative to Theia, i.e., the more negative the value, the better Theia performed relative to the alternative methods. A value greater than zero indicates that Theia was outperformed and zero indicates that both methods performed equally. In each case, the *x*-axis indicates the regulation strength and the *y*-axis indicates the relative false positive rate in the putative miRNA-gene interactions. Note that the *x*-axis stops at 0.15 for the F$^{1}$ comparison because all of the methods except Tiresias reached their maximum potential score at or before this point. The ARI for Tiresias cannot be calculated because Tiresias only predicts miRNA-gene interactions; thus, we plot the F$^{1}$ score a second time (bottom left) with a different axis to show Tiresias reaching its maximum potential F$^{1}$ score. In this plot, we see that Tiresias has a slight advantage over Theia in recovering interactions when the regulation strength is high. However, at a low regulation strength, which is biologically more relevant, Theia significantly outperforms Tiresias. We can also see that Theia achieves a significantly higher ARI than SNMNMF and PIMiM

We also compare Theia with SNMNMF, PIMiM, and Tiresias in terms of the F$^{1}$ score and ARI using the same synthetic data set, which we used to evaluate Theia. Figure 8 shows the results of each method relative to Theia. Theia outperforms SNMNMF and PIMiM in both ARI and F$^{1}$ score for all combinations of $p_\text {signal}$ and $p_\text {fp}$. We hypothesize that SNMNMF and PIMiM performed poorly during these ARI experiments because these methods were not tested with synthetic data during their formulation. We believe that the design and the tuned hyper-parameters of these methods overfit to the specific data sets on which they were originally fitted. Thus, they are unable to model different data sets as easily as Theia. Compared to Tiresias, Theia has a significant advantage when the signal strength ($p_\text {signal}$) is weak. We postulate that this stems from Theia’s novel use of ${\varvec{U}}$ and ${\varvec{V}}$ to disconnect non-interacting miRNA-gene pairs in the regression network.

**Fig. 9:**
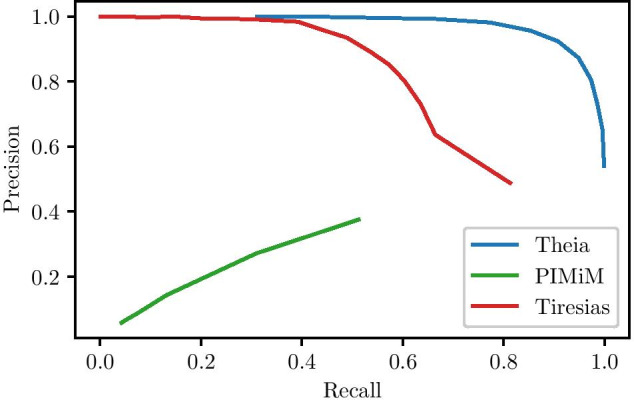
Precision-recall curve. Theia is able to achieve high recall without an uptick in the false positives. Tiresias on the other hand performs well at lower recall regions, while PIMiM suffers from a high level of false-positives. The AUPR (area under precision-recall curve) values for Theia, Tiresias and PIMiM are 0.91, 0.73 and 0.47 respectively

Figure 9 zooms in on one combination of $p_\text {signal}$ and $p_\text {fp}$ (1.0 and 0.1 respectively), and shows the precision-recall curve for Theia, PIMiM, and Tiresias. Because Theia is able to suppress noise in the miRNA-gene interaction matrix better than Tiresias, we minimize the number of false positive interactions, and thus we are able to push the threshold lower; by doing so, we greatly increase our recall without sacrificing much precision.

**Fig. 10:**
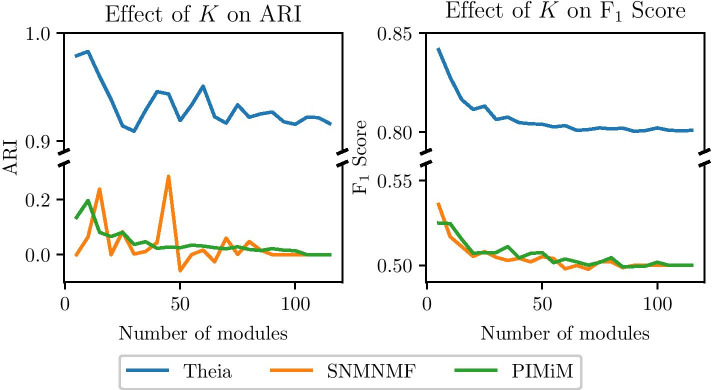
Effect of number of modules (the hyperparameter, *K*) on ARI and F$^{1}$ score. The ARI and F$^{1}$ score are plotted as a function of the number of modules (*x*-axis) for Theia (blue), SNMNMF (orange), and PIMiM (green). The number of miRNAs is five times the number of modules and the number of genes is fifty times the number of modules. Both the ARI and F1 score of Theia are significantly above those of the competing protocols for the entire range of the number of modules

Using the synthetic data set, we also study the effect of number of modules (*K*) on Theia, SNMNMF, and PIMiM. For this, we vary the value of *K*, and correspondingly the numbers of miRNAs (=5X number of modules) and genes (=50X number of modules) as well. The result of the ARI and F$^{1}$ score is shown in Fig. 10. We can see that both ARI and F$^{1}$ score change depending on *K*. However, in case of Theia, ARI and F$^{1}$ score stay above 0.9 and 0.8, respectively. In contrast, SNMNMF and PIMiM achieve much lower ARI and F$^{1}$ score, regardless of *K*. Note also that as the number of modules increases, the sparsity of the input matrices also increases, and this is characteristic that Theia handles particularly well.

## Visualizing Theia’s Results

**Fig. 11:**
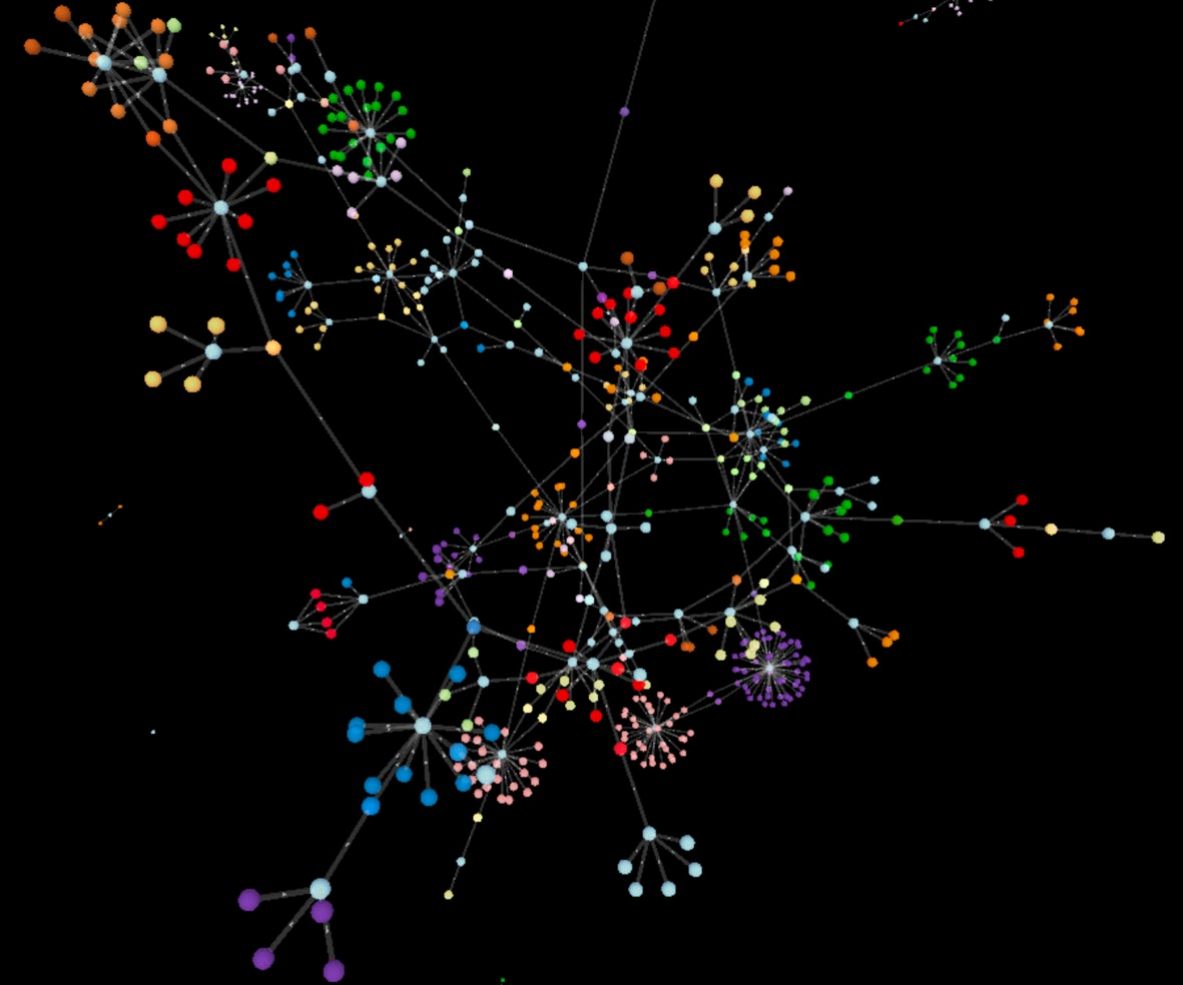
Three-dimensional visualization of a subset of the modules discovered by Theia

As part of our Theia framework, we also include a method of a visualizing the generated modules (See Fig. 11). Typical approaches-such as the one used in Le, et al. [20]-create a graph by connecting pairs of genes and pairs miRNAs and genes that interact.

The problem is that if such a graph is created with a non-trivial number of input miRNAs and genes, the result becomes unreadable. For this reason, we create one node for each comodule and point all the genes that are part of that comodue to it. If a gene is part of two modules than it will point to two modules and so on. We used the force-directed algorithm [76] to render the resulting graph. Our implementation is interactive and allows the user to rotate the graph, which means that overlapping nodes are less of a problem.

We can see from the visualization that the number of modules in which a particular gene is a member follows the skew normal distribution-the majority of genes only point to one module, a fair number point to two, and even fewer point to three, and so on. We also see the varying number of genes per module. A few modules are very large while the majority only contain a few genes.

## Discussion

In this paper, we have presented an algorithm called Theia that can predict the effects of modules of miRNAs on genes. Our work gets to the heart of the biological discovery that groups of miRNAs act combinatorially to regulate genes. Theia is a theoretically rigorous optimization algorithm that *simultaneously* predicts the strength and direction (i.e., up-regulation or down-regulation) of the effect of modules of miRNAs on a gene. We validated Theia by testing it on 1161 breast invasive carcinoma samples from the TCGA data portal, which contains 979 miRNAs and 19,258 genes. This resulted in the identification of 112 regulatory comodules. We found that some of the miRNA modules generated by our model are biologically significant (37 are enriched in spatial miRNA clusters and 48 have at least one enriched GO biological process), e.g., the miRNAs belonging to a given module are found to have similar functional roles, as determined by prior laboratory studies, or are proximate (prior studies have indicated that spatially clustered miRNAs often have similar functional roles). Similarly, we found that the gene modules are significantly enriched in many GO BP terms and form highly connected interaction networks. We posit that if Theia is capable of recovering known clusters of genes and miRNA, then the clusters found by our method *not* previously identified by literature are also likely to have biological significance. We believe that these novel regulatory comodules found by our method will be a springboard for further research into the specific functional roles of these new functional ensembles of miRNAs and genes, especially those related to diseases like breast cancer.

To further validate Theia, we generated synthetic data sets where the ground truth was known for all samples using parameters determined from real data. Notably, we found that the same hyperparameters of our model work well for both the real and synthetic data sets, indicating that our algorithmic framework is stable and robust to changes in the quality and type of input data. We evaluate the quality of miRNA-gene clustering and the accuracy of the interaction predictions obtained by Theia through comparison to prior works PIMiM, SNMNMF, and Tiresias. At a very high level of false positives ($p_\text {fp} = 2$, $p_\text {signal} = 0.5$), we see that Theia achieves an ARI score of 0.60 (2.9 times improvement over SNMNMF and PIMiM), and the F$^{1}$ score of 0.55 (1.9 times improvement over SNMNMF and PIMiM). When the signal strength is very low ($p_\text {signal} = 0.15$, $p_\text {fp} = 2$), Theia achieves an F$^{1}$ score of 0.55 (1.4 times improvement over Tiresias).

In future work, we are looking at modeling the effects of modules of miRNAs using a non-linear regression model. More substantively, we are looking at jointly modeling the effects of miRNAs, Transcription Factors, and *cis*-regulatory modules on gene expression levels. By considering the overall logic of gene expression profiles, we can holistically map out the gene regulatory networks and thus have a better handle on how to detect anomalies in gene expression in disease.

On the algorithmic front, we are looking at creating building blocks, which we call kernels, of genome annotating algorithmic motifs. These kernels can then be put together and optimized for specific end goals, rather than creating these kernels *de novo*, as outlined in our vision for the *Sarvavid* domain-specific language (DSL) framework [77]. For example, we will be augmenting our Theia framework by including additional trans-regulatory factors, such as TFs, plus additional cis-regulatory factors, such as DNA regulatory sequences [78], in an attempt to wholly map out the gene expression landscape. We have already seen a glimpse of this by augmenting Tiresias with the insight that genes and miRNA work together in many-to-many capacities, and in modules, in addition to acting individually. Our machine learning models will become progressively better as more biologically validated data becomes available, whether it is putative miRNA-gene interactions, their expression levels through mutual interactions, or protein-protein interaction data.

## Supplementary Information

**Additional file 1.** MiRNA-gene comodules.

**Additional file 2.** Comodule analysis.

**Additional file 3.** Synthetic data generation.

**Additional file 4.** Distribution of sizes of spatial miRNA clusters and GO terms.

## Data Availability

The tool is available at https://bitbucket.org/cellsandmachines/theia/. The datasets used and/or analysed during the current study are available from the corresponding author on reasonable request.
