# Supplementary material for: Simultaneous learning of individual microRNA-gene interactions and regulatory comodules
Source: BMC Bioinformatics. 2021 May 10;22:237. doi: 10.1186/s12859-021-04151-2 (PMC8111732; doi:10.1186/s12859-021-04151-2)
Supplement: Supplementary file 3 — Additional file 3. Synthetic data generation. [file 12859_2021_4151_MOESM3_ESM.pdf]

Synthetic data generation

For the detailed description of the synthetic generation algorithm, the following notations will be used to denote finite sequences and sequences of sequences respectively:

$$(a_i)_{i=1}^{n} = (a_1, a_2, \ldots, a_n)$$

$$((a_{i,j})_{i=1}^{n_j})_{j=1}^{m} = ((a_{1,1}, \ldots, a_{1,n_1}), (a_{2,1}, \ldots, a_{2,n_2}), \ldots, (a_{m,1}, \ldots, a_{m,n_n}))$$

For the following procedure, we will be using positive integers to represent distinct miRNA and genes. Also note that this procedure must be followed twice: once to generate the miRNA modules and a second time to generate the gene modules.

There are two distributions that must be considered when generating comodules. The first distribution is the number of modules sharing each miRNA or gene. The second distribution is the number of miRNAs or genes per module. These distributions are represented respectively by the D-dimensional vectors $\boldsymbol{d}$ and $\boldsymbol{d'}$, whose components are distributed according to the skew normal distribution, $\mathcal{SN}(\xi, \omega, \alpha)$, with location parameter ξ, scale parameter ω, and shape parameter α:

$$\begin{aligned} d_i &\sim |\lfloor \mathcal{SN}(1, 1, 5) \rfloor| \\ d'_i &\sim |\lfloor \mathcal{SN}(T/K, 10, 5) \rfloor|, \end{aligned}$$

where K is the number of modules and D is either the number of miRNAs (first time) or the number of genes (second time). The sequence (m_k), which is of length $L = \sum_{i=1}^{T} d_i$, represents the miRNAs or genes that will become module members. The number of times a particular miRNA or gene occurs in (m_k) follows the skew normal distribution, d_i. It is constructed by concatenating D sequences, each of which consist of the integer i repeated d_i times (representing repeated occurrences of the same miRNA or gene):

$$(m_k) = (\underbrace{1, 1, \ldots, 1}_{d_1 \text{ terms}}, \underbrace{2, 2, \ldots, 2}_{d_2 \text{ terms}}, \ldots, \underbrace{D, D, \ldots, D}_{d_D \text{ terms}})$$

After this, the terms of (m_k) are shuffled and the modules $(M_{i,1})_{i=1}^{d'_1}$ through $(M_{i,K})_{i=1}^{d'_K}$ are constructed by deriving K subsequences from (m_k). The lengths of these subsequences will be distributed according to the $\boldsymbol{d'}$ distribution. For each jth module $(M_{i,j})_{i=1}^{d'_j}$, we take the terms of (m_k) between the indices $c_j - d'_j + 1$ and $\min(c_j, L)$, inclusive, where $c_j = \sum_{k=1}^{j} d'_k$. However, if $c_j - d'_j + 1 > L$, the module shall be deleted. Thus, the actual number of modules generated may be less than K. Since in practice we cannot know the real number of modules, this was considered acceptable.

The entire process as described above is executed with $D = I$ (the number of miRNAs) and the miRNA-module-membership matrix, $\boldsymbol{U}$, is created by setting $u_{tj} = 1$ for each of the terms, t within each (jth) module, $(M_{i,j})_{i=1}^{d'_i}$. The remaining elements of $\boldsymbol{U}$ are zero. The process is executed a second time for $D = J$ (the number of genes) and the $\boldsymbol{V}$ matrix is initialized in the same fashion.
