# Supplementary figures and images for: Simultaneous learning of individual microRNA-gene interactions and regulatory comodules
Source: BMC Bioinformatics. 2021 May 10;22:237. doi: 10.1186/s12859-021-04151-2 (PMC8111732; doi:10.1186/s12859-021-04151-2)

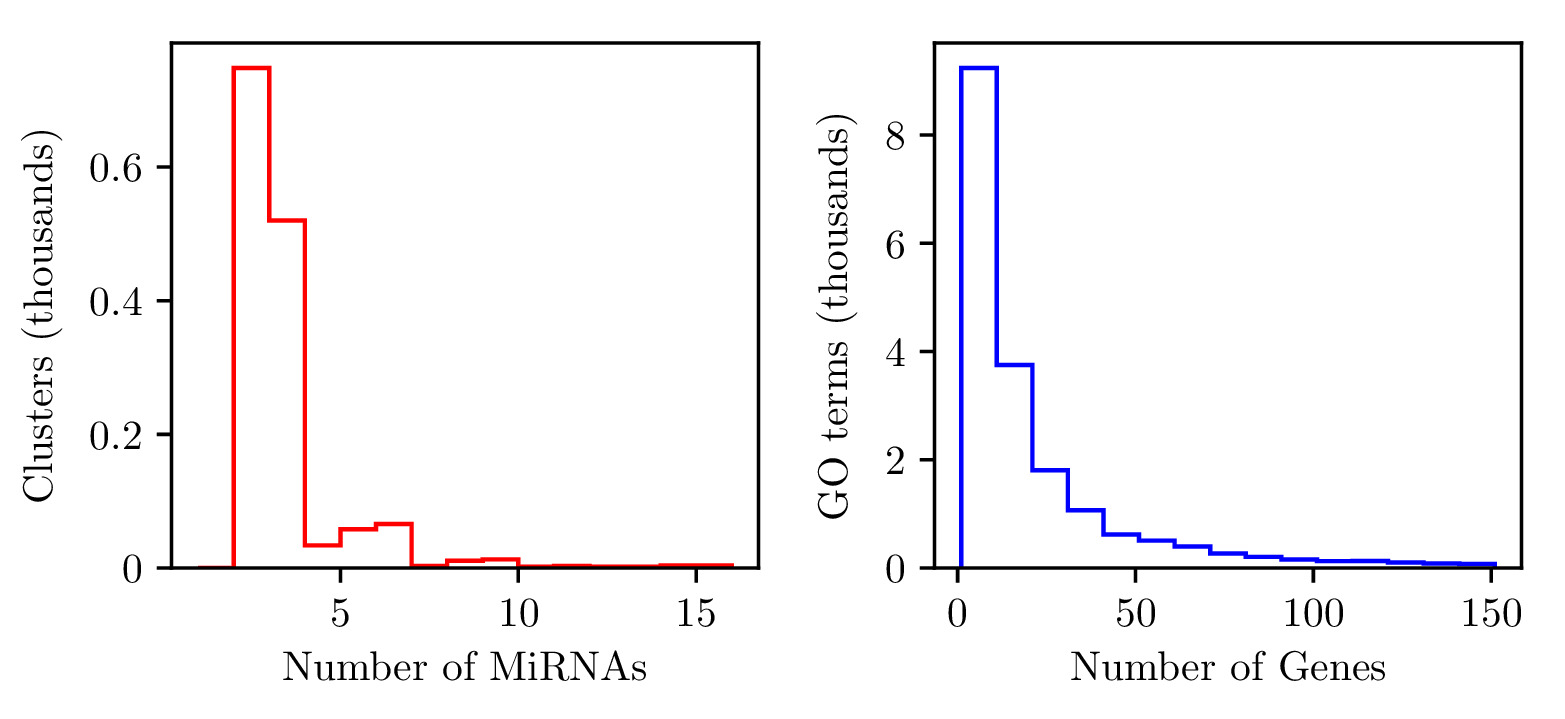

Supplement: Supplementary file 4 — Additional file 4. Distribution of sizes of spatial miRNA clusters and GO terms. [file 12859_2021_4151_MOESM4_ESM.jpg]
